# More than 50 long-term effects of COVID-19: a systematic review and meta-analysis

**DOI:** 10.1038/s41598-021-95565-8

**Published:** 2021-08-09

**Authors:** Sandra Lopez-Leon, Talia Wegman-Ostrosky, Carol Perelman, Rosalinda Sepulveda, Paulina A. Rebolledo, Angelica Cuapio, Sonia Villapol

**Affiliations:** 1 Drug Development, Novartis Pharmaceuticals, Florham Park, NJ USA; 2 Instituto Nacional de Cancerología, Subdirección de Investigación Básica, Mexico City, Mexico; 3 National Autonomous University of Mexico, SOMEDICyT, RedMPC, Mexico City, Mexico; 4 Harvard T.H. Chan School of Public Health, Boston, MA USA; 5 Divison of Infectious Diseases, Emory University School of Medicine, Atlanta, GA USA; 6 Hubert Department of Global Health, Rollins School of Public Health, Emory University, Atlanta, GA USA; 7 Center for Infectious Medicine, Department of Medicine Huddinge, Karolinska Institutet, Stockholm, Sweden; 8 Department of Neurosurgery, Center for Neuroregeneration, Houston Methodist Research Institute, 6670 Bertnet Avenue, Houston, TX 77030 USA; 9 Department of Neuroscience in Neurological Surgery, Weill Cornell Medical College, New York, USA

**Keywords:** Health care, Neurology, Signs and symptoms

## Abstract

COVID-19 can involve persistence, sequelae, and other medical complications that last weeks to months after initial recovery. This systematic review and meta-analysis aims to identify studies assessing the long-term effects of COVID-19. LitCOVID and Embase were searched to identify articles with original data published before the 1st of January 2021, with a minimum of 100 patients. For effects reported in two or more studies, meta-analyses using a random-effects model were performed using the MetaXL software to estimate the pooled prevalence with 95% CI. PRISMA guidelines were followed. A total of 18,251 publications were identified, of which 15 met the inclusion criteria. The prevalence of 55 long-term effects was estimated, 21 meta-analyses were performed, and 47,910 patients were included (age 17–87 years). The included studies defined long-COVID as ranging from 14 to 110 days post-viral infection. It was estimated that 80% of the infected patients with SARS-CoV-2 developed one or more long-term symptoms. The five most common symptoms were fatigue (58%), headache (44%), attention disorder (27%), hair loss (25%), and dyspnea (24%). Multi-disciplinary teams are crucial to developing preventive measures, rehabilitation techniques, and clinical management strategies with whole-patient perspectives designed to address long COVID-19 care.

## Introduction

The severe acute respiratory syndrome coronavirus 2 (SARS-CoV-2) was detected in China in December 2019. Since then, more than 175 million people worldwide have been infected after a year, and over 3.8 million people have died from the coronavirus disease 2019 (COVID-19)^1^. Although unprecedented efforts from the scientific and medical community have been directed to sequence, diagnose, treat, and prevent COVID-19, individuals' lasting effects after the acute phase of the disease are yet to be revealed.

The terminology has been confusing and not standardized. Different authors have used several terms to describe prolonged symptoms following COVID-19 illness, such as “Long COVID-19”, “post-acute COVID-19”, “persistent COVID-19 symptoms”, “chronic COVID-19”, “post-COVID-19 manifestations”, “long-term COVID-19 effects”, “post COVID-19 syndrome”, “ongoing COVID-19”, “long-term sequelae”, or “long-haulers” as synonyms. Most recently, the term “post-acute sequelae of SARS-CoV-2 infection” (PASC), “long-COVID-19”, and “post-acute COVID-19”, has been utilized^2^.

Symptoms, signs, or abnormal clinical parameters persisting two or more weeks after COVID-19 onset that do not return to a healthy baseline can potentially be considered long-term effects of the disease^3^. Although such alteration is mainly reported in severe and critical disease survivors, the lasting effects also occur in individuals with a mild infection who did not require hospitalization^4^. However, it has not yet been established how sex, gender, age, ethnicity, underlying health conditions, viral dose, or progression of COVID-19 significantly affect the risk of developing long-term effects of COVID-19^5^.

Since first reported, there has been a vast amount of social media patient groups, polls, comments, and scientific articles aiming to describe the chronicity of COVID-19. In parallel, hundreds of scientific publications, including cohorts studying specific effects of the disease and lists of case reports, have been described^6^. However, a broad overview of all the possible longstanding effects of COVID-19 is still needed. Therefore, our study aimed to perform a systematic review and meta-analysis of peer-reviewed studies to estimate the prevalence of all the symptoms, signs, or abnormal laboratory parameters extending beyond the acute phase of COVID-19 reported to date.

## Methods

### Database search strategy

The databases used to identify the studies were LitCOVID^7^, which includes all COVID articles in PubMed and Medline) and Embase. The studies classified in this meta-analysis included those published in the year 2020 (strictly before January 1st, 2021).

The search terms or keywords used were: (COVID-19) OR (COVID) OR (SARS-CoV-2) OR (coronavirus) OR (2019-nCoV) AND (long* OR haulers OR post OR chronic OR term OR complications OR recurrent OR lingering OR convalescent OR convalescence OR persist*. Given that LitCOVID includes all articles from MedLine, in the search in Embase, we excluded the articles from MedLine and those not related to COVID-19. The systematic review followed the Preferred Reporting Items for Systematic Reviewers and Meta-analysis (PRISMA) guidelines^8,9^. The registration of the review protocol was not previously done.

### Inclusion and exclusion criteria

The inclusion criteria of the search were as follows: (1) to identify peer-reviewed human studies in English that reported symptoms, signs, or (2) laboratory parameters of patients at a post-COVID-19 stage (assessed 2 weeks or more after initial symptoms) in cohorts of COVID-19 patients. All types of studies, including randomized controlled trials, cohorts, and cross-sectional studies, were analyzed only when the cases (numerator) were part of a COVID-19 cohort (denominator). Titles, abstracts, and full texts of articles were independently screened by two authors (S.L.L. and T.W.O.). The complete article was reviewed in case of a difference of opinion on the inclusion based on title or abstract. Disagreement on the inclusion of a full-text article was discussed with all the authors. We exclude letters, editorials, reviewers, and commentaries. The exclusion criteria were: (1) not written in English; (2) have less than 100 patients included in the study. To estimate the prevalence of long-term erects in patients with COVID-19, we needed to include as a denominator the patients with acute COVID-19 (with and without long-term effects). Therefore, it is not possible to include case studies (usually less than 100 persons). The larger the denominator, the greater the reliability and generalizability of the estimate, and the lower the possibility of bias of including only patients that developed long COVID-19. We also exclude non-English language studies due to a lack of robust resources for accurate translation.

### Data extraction and analysis

Data were extracted by five review authors (C.P., A.C., P.R., R.S., S.V.), and each study's quality was assessed using the Health States Quality-Controlled data (QCed) by two review authors independently (S.L.L. and T.W.O.). This index is described and recommended by the MetaXL Guidelines. It is specific to evaluate the quality of studies assessing prevalence. Relevant studies were then subjected to full-text screening by the same reviewers. The descriptive variables extracted were country, setting, follow-up time, the severity of COVID-19, sample size, mean age and percentage of gender, outcomes, and names used to describe the long-term effects of COVID-19 (Supplemental Table S1).

### Outcomes

All the diseases, disorders, symptoms, signs, and laboratory parameters reported total numbers or percentages were included. Outcomes of interest were blood biomarkers and abnormal chest X-ray/CT reported for patients with SARS-CoV-2 infection in any setting. In addition, we assessed symptoms in several distinct systems; neurological, respiratory, gastrointestinal, cardiac, endocrine, dermatological, hepatic, and renal. When two time points were reported in the study, the outcomes assessed after the most extended follow-up were used.

### Statistical analysis

For effects reported only in a single study, the prevalence was estimated by dividing the number of patients with each symptom by the total number of COVID-19 patients in the sample multiplied by 100 to calculate the percentage. For effects reported in two or more studies, meta-analyses using a random-effects model were performed using the MetaXL software to estimate the pooled prevalence, which uses a double arcsine transformation^10^. Prevalence with 95% confidence intervals (CI) was presented. Heterogeneity was assessed using *I*^2^ statistics. The Preferred Reporting Items for Systematic Reviewers and Meta-analysis (PRISMA) 2020 guideline was followed. Given the heterogeneity expected, a random-effects model was used. Heterogeneity was assessed using the *I*^2^ statistics. Values of 25%, 50%, and 75% for *I*^2^ represented low, medium, and high heterogeneity. Sensitivity analyses were performed to assess the contribution of each study. Although none of the included definitions, or effects, were pre-specified, all of the effects and definitions were determined via each identified study. Publication bias in the selected study was evaluated by plotting the funnel plot and subsequent analyses. Each study's quality was assessed and described using the MetaXL Guidelines, which is specific to assess the quality of studies assessing prevalence. A description of what was considered is found in Supplemental Table S1.

## Results

**Figure 1:**
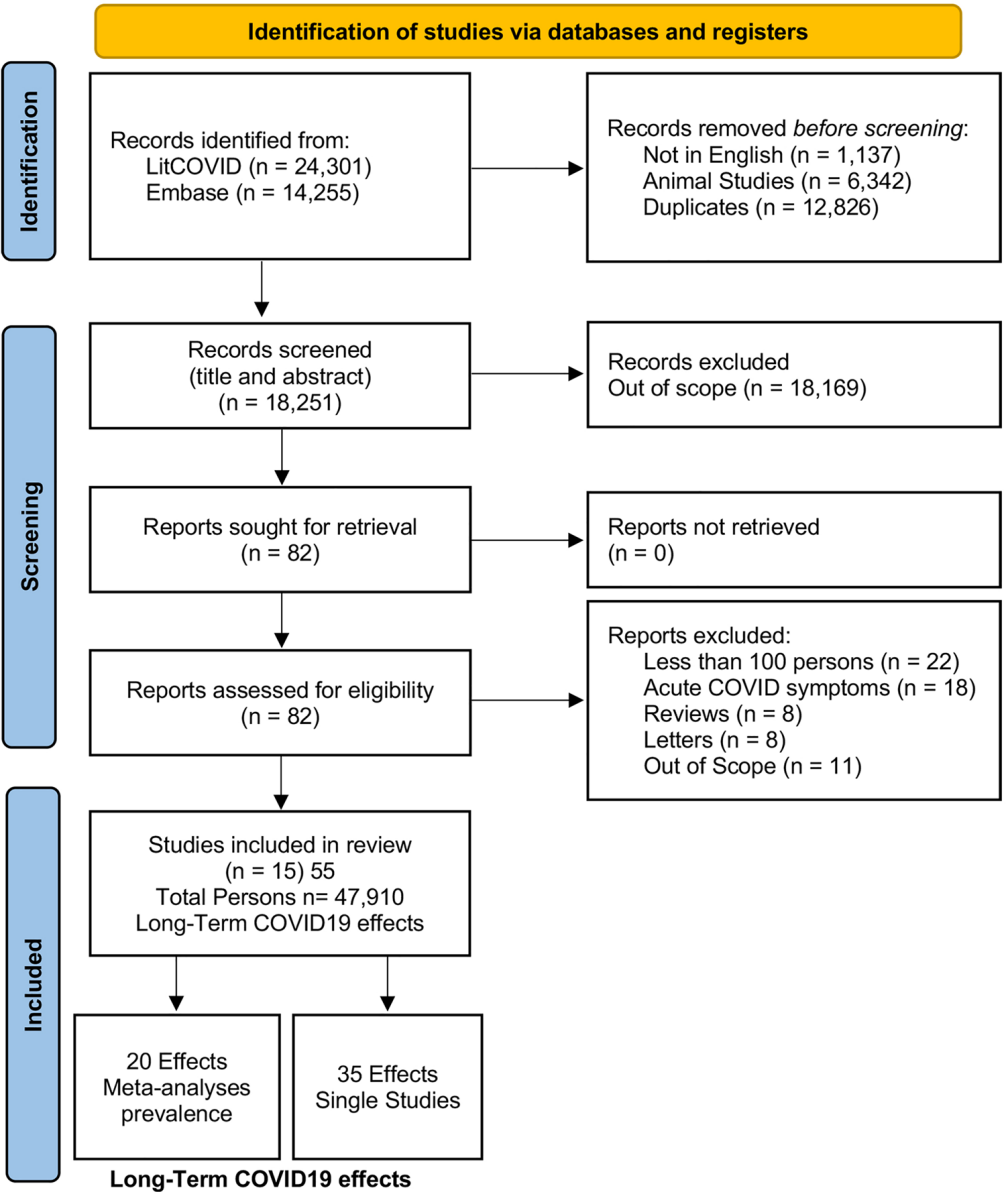
Study selection. Preferred items for Systematic Reviews and Meta-Analyses (PRISMA) flow diagram. Out of 15,917 identified studies and after application of the inclusion and exclusion criteria, 15 studies were included in the quantitative synthesis.

The title and abstract of 18,251 publications were screened. Of these, 82 full publications were reviewed for removal of duplication and initial eligibility assessment of title/abstract of all articles based on the eligibility criteria. Nineteen studies were excluded because they involved less than 100 persons. Thus, a total of 15 studies were selected to be analyzed. The process of study selection is presented in Fig. 1.

### Characteristics of the included studies

There were eight studies from Europe and UK, three from the USA, one from Australia, China, Egypt, and Mexico (Table 1). The number of patient cohorts that were followed up in the studies ranged from 102 to 44,799. Adults ranging from 17 to 87 years of age were included. The patient follow-up time ranged from 14 to 110 days. Ten studies collected information from the patients using self-reported surveys. Two studies collected data from medical records and three by a clinical evaluation. Six out of the 15 studies included only patients hospitalized for COVID-19. The rest of the studies mixed with mild, moderate, and severe COVID-19 patients. There were no studies with overlapping samples. Two meta-analyses showed low heterogeneity (*I*^2^ < 25%), two showed medium heterogeneity, and the rest had high (*I*^2^ > 75%).

**Table 1:** Characteristics of all included studies.

Author^ref^	Country	Setting	Follow-up timepoint mean	Population	Sample size (n)	Age mean (SD)/range	Sex % male	Outcomes	Term used to refer to long-term effects
Andrews^11^	UK, Italy	Multicenter, validated survey	52 days	Mild to moderate health care workers	114	Median 38	24.6	Hyposmia, anosmia, hypogeusia, ageusia, dysgeusia	NR
Carfì^12^	Italy	Single center, clinical and survey	60 days	Hospitalized	143	56.5 (19–84)	63	Fatigue, dyspnea, joint pain, chest pain, cough, anosmia, Sicca syndrome, Rhinitis, red eyes, dysgeusia, headache, sputum, lack of appetite, sore throat, vertigo, myalgia, diarrhea	Persistent symptoms Post-acute COVID-19
Carvalho-Schneider^13^	France	University hospital, phone survey	60 days	Mild, moderate, and severe	150	49 (44–64)	44	Weight loss > 5%, severe dyspnea or asthenia, asthenia, chest pain, palpitations, anosmia/ageusia, headache, cutaneous signs, arthralgia, myalgia, digestive disorders, fever, sick leave	Symptom persistence
Chopra^14^	USA	Multicenter, medical records	60 days	Hospitalized, and ICU	488	62	51.8	Persistent symptoms and New symptoms: Anosmia, dysgeusia, cough, shortness of breath/chest tightness/wheezing, chest problems, breathlessness, oxygen use, new use of CPAP or another breathing machine when asleep emotional impact (50%) and (financial impact)	Long term sequelae
Galvan-Tejada^15^	Mexico	Questionnaire in 3 cities, survey	31 days	NA	141	39	49	Chills, dyspnea, anosmia, dysgeusia, nausea or vomiting, cough, red eyes	Persistent symptoms
Garrigues^16^	France	Single center, validated surveys	110 days	Hospitalized and ICU	120	63.2	62.5	Cough, chest pain, fatigue, dyspnea, ageusia, anosmia, hair loss, attention disorder, memory loss, sleep disorder	Post-discharge symptoms
Horvath^17^	Australia	Health database, survey	83 days	Mild, moderate	102	45 (17–87)	40	Anosmia, ageusia, hyposmia, hypogeusia	Post-recovery
Kamal^18^	Egypt	General population, survey	NR	80% mild 15% moderate 5% severe ICU	287	32.3 (20–60)	35.9	Fatigue, anxiety, joints pain, continuous headache, chest pain, dementia, depression. Dyspnea, blurred vision, tinnitus, intermittent fever, obsessive–compulsive disorder, pulmonary fibrosis, diabetes mellitus, migraine, stroke, renal failure, myocarditis, arrhythmia	Post- COVID-19 manifestations
Mandal^19^	UK	3 hospitals, survey	Median 54 days	59% oxygen 14.5% ICU 7.1% intubation 26% mild 41% moderate 30% severe	384	59.9 (± 16.1)	62	Breathlessness, cough, fatigue, depression, elevated D-dimer, elevated C reactive protein, abnormal chest radiograph, poor sleep quality	Long-COVID
Munro^20^	UK	University hospitals, clinical	8 weeks	Hospitalized	121	64 (44–82)	87.5	Changes in hearing, tinnitus	Persistent
Sonnweber^21^	Austria	Multicenter, clinical and laboratory	100 days	75% Hospitalized 50% oxygen 25% outpatient Mild (N = 36), moderate (N = 37), severe (N = 40), critical (N = 32)	145 and 135	57 (50–70)	55	Dyspnea, cough, fever, diarrhea, vomiting, pain, night sweat, sleep disorder, hyposmia/anosmia, reduced lung diffusing capacity, CT lung abnormalities, CRP, IL-6, PCT, D-dimer, nt-PRObnp, serum ferritin	Persistent symptoms Long-term sequelae
Taquet^22^	USA	Electronic health records, electronic health records	Range 14–90 days	No previous history of psychiatric disorders	44,779	49.3 (19.2)	45.1	New: psychiatric illness disorders psychotic, insomnia, mood disorders (depressive episodes), anxiety disorders (PTSD, panic disorder, adjustment disorder, and generalized anxiety disorder)	COVID-19 sequela
Tenforde^3^	USA	CDC multistate telephone interview nationwide, survey	Range 14–21 days	Symptomatic outpatient	270	18–50	48	Vomiting, confusion, abdominal pain, chest pain, sore throat, nausea, dyspnea, congestion, diarrhea, loss of smell, loss of taste, chills, fever, body aches, headache, cough, fatigue	Prolonged symptoms Prolonged illness
Townsend^23^	Ireland	Outpatient clinic, validated survey	Range 56 days–12 weeks	Mild, moderate symptomatic, outpatient, and 55.5% hospital	128	49.5	46.1	Fatigue (only symptoms studied)	Persistent fatigue
Xiong^24^	China	Single center, survey	97 days	Hospitalized	538	52 (41–62)	45.5	General symptoms, physical decline/fatigue, post-activity polypnoea, respiratory, cardiovascular, psychosocial, alopecia	Clinical sequelae

*NR* not reported.

The populations were well defined. However, most studies were mixed mild, moderate, and severe patients—none of the studies were stratified by different by severity. The observation period was also well defined. However, none of the studies presented their results as stratified by observation. Therefore, it was impossible to identify the source of heterogeneity and it was not possible to assess how long each symptom lasted. Seven of the studies did not describe the system used to record the symptoms in detail, and most were self-reported retrospectively. A high score was given to the studies that administered an interview, included multi-sites surveys, and reported point prevalence. All of the studies received a score of greater than 8 (out of 11 points).

### Abnormalities of laboratory parameters

**Figure 2:**
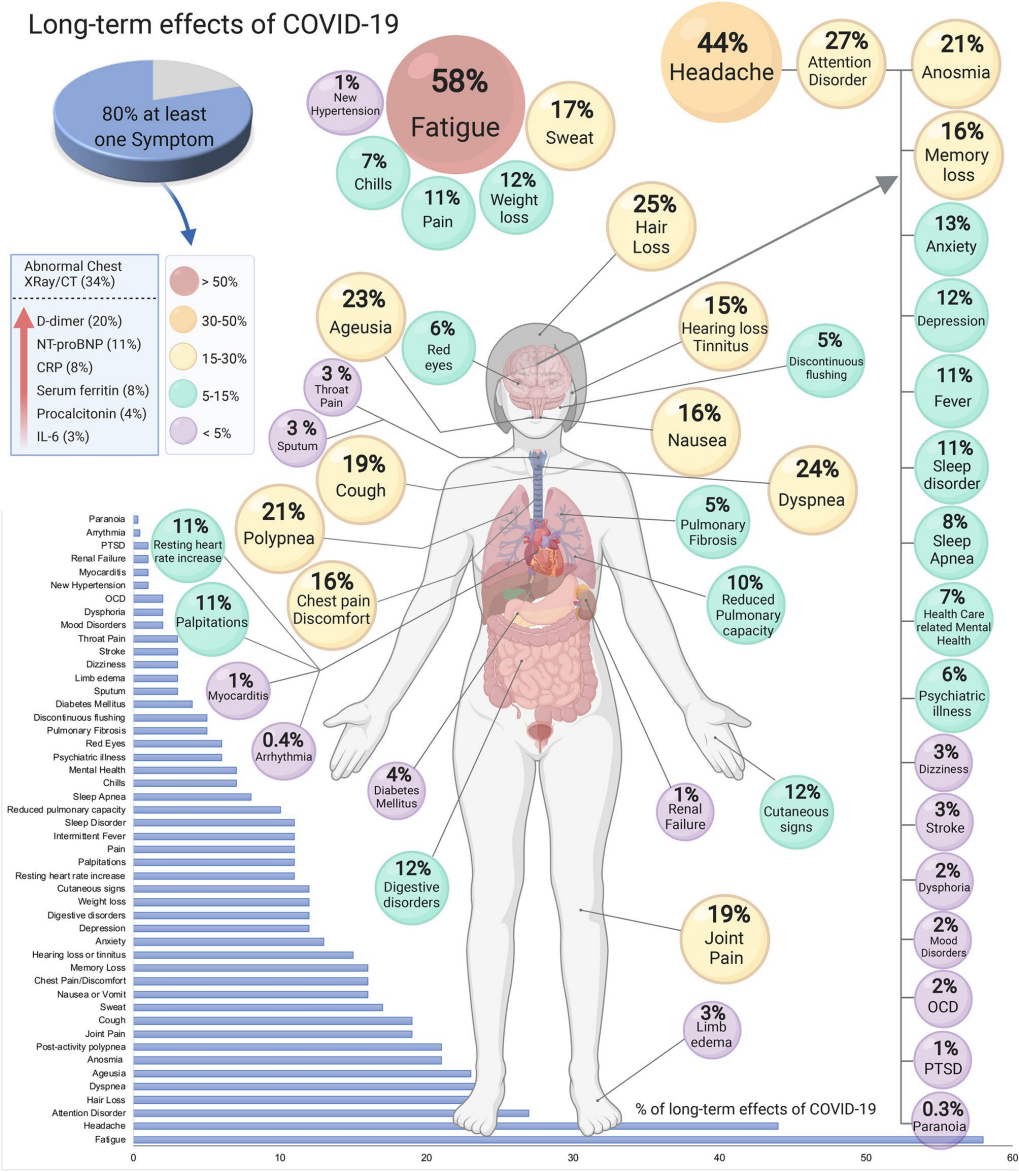
Long-term effects of coronavirus disease 2019 (COVID-19). The meta-analysis of the studies included an estimate for one symptom or more reported that 80% of the patients with COVID-19 have long-term symptoms. *CRP* C-reactive protein, *CT* computed tomography, *IL-6* Interleukin-6, *NT-proBNP* (NT)-pro hormone BNP, *OCD* Obsessive Compulsive Disorder, *PTSD* Post-traumatic stress disorder. This figure was created using Biorender.com.

Measurable parameters included 6 elevated laboratory parameters. An abnormal chest X-ray/CT was observed in 34% (95% CI 27–42) of the patients. Markers reported to be elevated were D-dimer (20%, 95% CI 6–39), N-terminal (NT)-pro hormone BNP (NT-proBNP), (11%, 95% CI 6–17), C-reactive protein (CRP) (8%, 95% CI 5–12), serum ferritin (8% 95% CI 4–14), procalcitonin (4% 95% CI 2–9) and interleukin-6 (IL-6) (3% 95% CI% 1–7) (Table 2, Fig. 2).

**Table 2:** Long-term effects in PASC patients.

	Studies	Cases	Sample size	Prevalence % (95% CI)
**Clinical manifestations**				
1 or > symptoms	7	1403	1915	80 (65–92)
Fatigue	7	1042	1892	58 (42–73)
Headache	2	261	579	44 (13–78)
Attention disorder	1	32	120	27 (19–36)
Hair loss	2	178	658	25 (17–34)
Dyspnea	9	584	2130	24 (14–36)
Ageusia	4	108	466	23 (14–33)
Anosmia	6	210	1110	21 (12–32)
Post-activity polypnea	1	115	538	21 (18–25)
Joint pain	4	191	1098	19 (7–34)
Cough	7	465	2108	19 (7–34)
Sweat	2	144	638	17 (6–30)
Nausea or vomit	1	22	141	16 (10–23)
Chest pain/discomfort	6	264	1706	16 (10–22)
Memory loss	3	320	45,186	16 (0–55)
Hearing loss or tinnitus	2	64	425	15 (10–20)
Anxiety	4	2288	45,896	13 (3–26)
Depression	4	182	1501	12 (3–23)
Digestive disorders	1	15	130	12 (7–18)
Weight loss	1	15	130	12 (7–18)
Cutaneous signs	1	15	130	12 (7–18)
Resting heart rate increase	1	60	538	11 (9–14)
Palpitations	1	14	130	11 (6–17)
General pain	1	17	145	11 (7–18)
Intermittent fever	1	32	287	11 (8–15)
Sleep disorder	5	1036	46,070	11 (3–24)
Reduced pulmonary diffusing capacity	1	14	145	10 (6–16)
Sleep apnea	1	34	404	8 (6–12)
Chills	2	44	679	7 (1–18)
Health care related mental health	1	28	404	7 (5–10)
Psychiatric illness	1	2597	44,779	6 (6–6)
Red eyes	1	8	141	6 (3–11)
Pulmonary fibrosis	1	14	287	5 (3–8)
Discontinuous flushing	1	26	538	5 (3–7)
Diabetes mellitus	1	12	287	4 (2–7)
Sputum	1	16	538	3 (2–5)
Limb edema	1	14	538	3 (1–4)
Dizziness	1	14	538	3 (1–4)
Stroke	1	8	287	3 (1–5)
Throat pain	1	17	538	3 (2–5)
Mood disorders	1	896	44,779	2 (2–2)
Dysphoria	1	9	538	2 (1–3)
Obsessive compulsive disorder (OCD)	2	15	579	2 (0–8)
New hypertension	1	7	538	1 (1–3)
Myocarditis	1	4	287	1 (0–4)
Renal failure	1	4	287	1 (0–4)
Post-traumatic stress disorder (PTSD)	1	2	292	1 (0–2)
Arrythmia	1	1	287	0.4 (0–2)
Paranoia	1	1	292	0.3 (0–2)
**Lab tests and other examinations**				
Abnormal chest X-ray/CT	2	188	529	34 (27–42)
Elevated D-dimer	2	134	529	20 (6–39)
Elevated NT-proBNP	1	16	145	11 (6–17)
Elevated C-reactive protein	2	44	529	8 (5–12)
Elevated serum ferritin	1	12	145	8 (4–14)
Elevated procalcitonin	1	6	145	4 (2–9)
Elevated IL-6	1	4	145	3 (1–7)

Random effects weighted by quality effects model MetaXL for 2 or more studiesC-reactive protein (CRP), Interleukin-6 (IL-6), D-dimer, NT-proBNP, serum ferritin, N-terminal (NT)-pro hormone BNP (NT-proBNP).

### Prevalence of long-term effects in COVID-19 patients

We identified a total of 55 long-term effects associated with COVID-19 in the literature reviewed (Table 2). Most of the effects correspond to clinical symptoms such as fatigue, headache, joint pain, anosmia, ageusia, etc. In addition, diseases such as stroke and diabetes mellitus were also present. Table 2 presents the prevalence of all the effects that were reported. It was possible to perform 21 meta-analyses. For the rest, the prevalence was estimated using one cohort. The meta-analysis of the studies (n = 7) that included an estimate for one symptom or more reported that 80% (95% CI 65–92) of the patients with COVID-19 have long-term symptoms.

### Overall prevalence of most common symptoms

The 5 most common manifestations were fatigue (58%, 95% CI 42–73), headache (44%, 95% CI 13–78), attention disorder (27% 95% CI 19–36), hair loss (25%, 95% CI 17–34), dyspnea (24%, 95% CI 14–36) (Table 2, Fig. 2). Other symptoms were related to lung disease (cough, chest discomfort, reduced pulmonary diffusing capacity, sleep apnea, and pulmonary fibrosis), cardiovascular (arrhythmias, myocarditis), neurological (dementia, depression, anxiety, attention disorder, obsessive–compulsive disorders), and others were unspecific such as hair loss, tinnitus, and night sweat (Table 2, Fig. 2, Supplemental Figure S1). A couple of studies reported that fatigue was more common in females, and one study reported that post-activity polypnea and alopecia were more common in females^4,24^. The rest of the studies did not stratify their results by age or sex.

## Discussion

This systematic review and meta-analysis shows that 80% (95% CI 65–92) of individuals with a confirmed COVID-19 diagnosis continue to have at least one overall effect beyond 2 weeks following acute infection. In total, 55 effects, including symptoms, signs, and laboratory parameters, were identified, with fatigue, anosmia, lung dysfunction, abnormal chest X-ray/CT, and neurological disorders being the most common (Table 1, Fig. 2). Most of the symptoms were similar to the symptomatology developed during the acute phase of COVID-19. However, given that all of the surveys were predefined, there is a possibility that other effects have not yet been identified. In the following paragraphs, we will discuss the most common symptoms to illustrate how complex each one can be. However, further studies are needed to understand each symptom separately and in conjunction with the other symptoms. The five most common effects were fatigue (58%), headache (44%), attention disorder (27%), hair loss (25%), and dyspnea (24%). The recovery from COVID-19 should be more developed than checking for hospital discharge or testing negative for SARS-CoV-2 or positive for antibodies^25^.

Fatigue (58%) is the most common symptom of long and acute COVID-19^23^. It is present even after 100 days of the first symptom of acute COVID-19^4,23^. In addition, there are syndromes such as acute respiratory distress syndrome (ARDS), in which it has been observed that after a year, more than two-thirds of patients reported clinically significant fatigue symptoms^26^. The symptoms observed in post-COVID-19 patients, resemble in part the chronic fatigue syndrome (CFS), which includes the presence of severe incapacitating fatigue, pain, neurocognitive disability, compromised sleep, symptoms suggestive of autonomic dysfunction, and worsening of global symptoms following minor increases in physical and/or cognitive activity^27–31^. Myalgic encephalomyelitis (ME) or CFS is a complex and controversial clinical condition without established causative factors, and 90% of ME/CFS has not been diagnosed^32^. Possible causes of CFS include viruses, immune dysfunction, endocrine-metabolic dysfunction, and neuropsychiatric factors. The infectious agents related to CFS have been Epstein-Barr virus, cytomegalovirus, enterovirus, and herpesvirus^33^. It is tempting to speculate that SARS-CoV-2 can be added to the viral agents' list causing ME/CFS.

Several neuropsychiatric symptoms have been reported, headache (44%), attention disorder (27%), and anosmia (21%). Other symptoms were reported, which were not included in the publications, including brain fog and neuropathy^34,35^. The etiology of neuropsychiatric symptoms in COVID-19 patients is complex and multifactorial. They could be related to the direct effect of the infection, cerebrovascular disease (including hypercoagulation)^36^, physiological compromise (hypoxia), side effects of medications, and social aspects of having a potentially fatal illness^37^. Adults have a double risk of being newly diagnosed with a psychiatric disorder after the COVID-19 diagnosis^37^, and the most common psychiatric conditions presented were anxiety disorders, insomnia, and dementia. Sleep disturbances might contribute to the presentation of psychiatric disorders^38^. Prompt diagnosis and intervention of any neuropsychiatric care is recommended for all patients recovering from COVID-19. An increase in mental health attention models in hospitals and communities is needed during and after the COVID-19 pandemic. Hair loss after COVID-19 could be considered as telogen effluvium, defined by diffuse hair loss after an important systemic stressor or infection. Premature follicular transitions cause it from the active growth phase (anagen) to the resting phase (telogen). It is a self-limiting condition that lasts approximately 3 months, but it could cause emotional distress^39^.

Dyspnea and cough were found in 24% and 19% of patients, respectively (Table 2, Fig. 2). In addition, abnormalities in CT lung scans persisted in 35% of patients even after 60–100 days from the initial presentation. In a follow-up study conducted in China among non-critical cases of hospitalized patients with COVID-19, radiographic changes persisted in nearly two-thirds of patients 90 days after discharge^40^. Although most of the available studies do not include baseline pulmonary dysfunction or radiographic abnormalities, findings indicate improvement or resolution of abnormal CT findings. Previous data from recovered patients with other viral pneumonia^41,42^, also found residual radiographic changes. Abnormalities in pulmonary function, such as decreased diffusion capacity for carbon monoxide, were present among 10% of patients in this meta-analysis. Although these findings are not as high as compared to other available studies of survivors with COVID-19 or SARS, where the estimate of lung dysfunction is 53% and 28% respectively^43,44^, the reasons behind these differences could be distinct follow-up periods, definitions of pulmonary dysfunction, or characteristics of the patient population. Nevertheless, residual radiographic findings or lung function abnormalities require additional investigation on their clinical relevance and long-term consequences.

The immune-mediated tissue damage in COVID-19 involves cellular and humoral responses, but the immunity to SARS-CoV-2 and the protection to reinfection or a final viral^40,45^ clearance is unknown. Also, the reason why some patients experience long-term symptoms after COVID-19 is uncertain. This could be partially explained by host-controlled factors that influence the outcome of the viral infection, including genetic susceptibility, age of the host when infected, dose and route of infection, induction of anti-inflammatory cells and proteins, presence of concurrent infections, past exposure to cross-reactive agents, etc. Whether SARS-CoV-2 can cause substantial tissue damage leading to a chronic form of the disease such as the chronic lesions in convalescence observed in other viruses such as human immunodeficiency virus (HIV), hepatitis C virus (HCV), hepatitis B virus (HBV), and some herpesviruses is still unknown.

One study was excluded because it did not provide a denominator, and therefore it was not possible to estimate the prevalence^46^. In such a study, the authors performed a survey in a Facebook group of patients who previously had COVID-19 and compared the symptoms of those hospitalized with mild to moderate symptoms. They concluded that both groups had symptoms after 3 months of having COVID-19. Symptoms that were not mentioned in any of the articles we studied include sudden loss of body weight, ear pain, eye problems, sneezing, cold nose, burning feeling in the trachea, dizziness, heart palpitations, pain/burning feeling in the lungs, pain between the shoulder blades, Sicca syndrome, vertigo, body aches, and confusion^3,12^.

The results assessed in the present study are in line with the current scientific knowledge on other coronaviruses, such as those producing SARS and MERS, both clinical sharing characteristics with COVID-19, including post symptoms. For example, studies on SARS survivors have shown lung abnormalities months after infection. After a 1-year follow-up, a study showed that 28% of the survivors presented decreased lung function and pulmonary fibrosis signs^44,47,48^. In addition, MERS survivors showed pulmonary fibrosis (33%)^49^. Regarding psychiatric symptoms, a study reported high levels of depression, anxiety, and post-traumatic stress disorder (PTSD)^37^ in the long term in patients previously infected with other coronaviruses.

To assure that future healthcare providers, researchers, and educators recognize the effects of long-term COVID19 that are sex- and age-specific related, it is essential to classify the groups according to such variables to make better decisions about prevention diagnosis and disease management.

Limitations of this systematic review and meta-analyses include the small sample size for some outcomes, making it difficult to generalize these results to the general population. The variation in the definition of some outcomes and markers and the possibility of bias. For example, several studies that used a self-reported questionnaire could result in reporting bias. In addition, the studies were very heterogeneous, mainly due to the follow-up time references and the mixture of patients who had moderate and severe COVID-19. All of the studies assessed had performed their internal pre-definition of symptoms, and therefore there is the possibility that essential outcomes were not reported. Another limitation is that, given that COVID-19 is a new disease, it is impossible to determine how long these effects will last. To decrease heterogeneity and better understand the long-term effects of COVID-19, there is a need for studies to stratify by age, previous comorbidities, the severity of COVID-19 (including asymptomatic), and the duration of each symptom. To determine whether these long-term effects either complicate previous diseases or continue COVID-19, there is a need for prospective cohort studies. The baseline characteristics should be well established. To obtain more accurate meta-analyses, there is an urgent need to have a standard definition of long-COVID-19. Currently, post-COVID-19 symptoms that develop during or after COVID-19 are defined if they continue for ≥ 12 weeks (“long-COVID-19”), and not explained by an alternative diagnosis^2,6,50^. There is a need to standardize biological measures such as peripheral blood markers of genetic, inflammatory, immune, and metabolic function to compare studies. Besides studying pre-defined symptoms and characteristics, an open question should be included. Proper documentation in medical charts by health care providers and the flexibility and collaboration from the patients to report their symptoms are of equal importance.

## Conclusion

More evidence and research from multi-disciplinary teams are crucial to understanding the causes, mechanisms, and risks to develop preventive measures, rehabilitation techniques, and clinical management strategies with whole-patient perspectives designed to address the after-COVID-19 care. There is a need for more information about prospective studies to better evaluate the natural course of COVID-19 infection and define the long- COVID-19 syndrome. From the clinical point of view, physicians should be aware of the symptoms, signs, and biomarkers present in patients previously affected by COVID-19 to promptly assess, identify and halt long COVID-19 progression, minimize the risk of chronic effects help reestablish pre-COVID-19 health. Management of all these effects requires further understanding to design individualized, dynamic cross-sectoral interventions in Post-COVID-19 clinics with multiple specialties, including graded exercise, physical therapy, frequent medical evaluations, and cognitive behavioral therapy when required^51,52^.

## Supplementary Information

Supplementary Information 1.

Supplementary Information 2.

Supplementary Information 3.

## Data Availability

All data relevant to the study are included in the article or uploaded as supplementary information. In addition, the datasets used and/or analyzed during the current study are available from the corresponding author on reasonable request.

## Abbreviations

ARDS: Acute respiratory distress syndrome
CI: Credible interval
COVID-19: Coronavirus disease 2019
CFS: Chronic fatigue syndrome
CRP: C-reactive protein
CT: Computed tomography
HIV: Human immunodeficiency virus
HCV: Hepatitis C virus
HBV: Hepatitis B virus
IL-6: Interleukin-6
NT: N-terminal
NT-proBNP: Pro hormone BNP
ME: Myalgic encephalomyelitis
MERS: Middle East respiratory syndrome
OCD: Obsessive Compulsive Disorder
PTSD: Post-traumatic stress disorder
SARS-CoV-2: Severe acute respiratory syndrome coronavirus 2
SARS: Severe acute respiratory syndrome
PTSD: Post-traumatic stress disorder

## References

[1] Ritchie, H., Ortiz-Ospina, E., Beltekian, D., Mathieu, E., Hasell, J., Macdonald, B., Giattino, C., Appel, C., Rodés-Guirao, L., & Roser, M. Coronavirus Pandemic (COVID-19). (2021).

[2] Rubin R (2020). As their numbers grow, COVID-19 "long haulers" stump experts. JAMA.

[3] Tenforde MW (2020). Symptom duration and risk factors for delayed return to usual health among outpatients with COVID-19 in a multistate health care systems network—United States, March-June 2020. Morb. Mortal Wkly Rep..

[4] Townsend L (2021). Persistent poor health post-COVID-19 is not associated with respiratory complications or initial disease severity. Ann. Am. Thorac. Soc..

[5] Gemelli Against C-P-ACSG (2020). Post-COVID-19 global health strategies: The need for an interdisciplinary approach. Aging Clin. Exp. Res..

[6] Greenhalgh T, Knight M, A'Court C, Buxton M, Husain L (2020). Management of post-acute covid-19 in primary care. BMJ.

[7] Chen Q, Allot A, Lu Z (2021). LitCovid: An open database of COVID-19 literature. Nucleic Acids Res..

[8] Shamseer L (2015). Preferred reporting items for systematic review and meta-analysis protocols (PRISMA-P) 2015: Elaboration and explanation. BMJ.

[9] Moher D (2015). Preferred reporting items for systematic review and meta-analysis protocols (PRISMA-P) 2015 statement. Syst. Rev..

[10] Barendregt JJ, Doi SA, Lee YY, Norman RE, Vos T (2013). Meta-analysis of prevalence. J. Epidemiol. Community Health.

[11] Andrews PJ (2020). Olfactory and taste dysfunction among mild-to-moderate symptomatic COVID-19 positive health care workers: An international survey. Laryngosc. Investig. Otolaryngol..

[12] Carfi A, Bernabei R, Landi F, Gemelli Against C-P-ACSG (2020). Persistent symptoms in patients after acute COVID-19. JAMA.

[13] Carvalho-Schneider C (2020). Follow-up of adults with noncritical COVID-19 two months after symptom onset. Clin. Microbiol. Infect..

[14] Chopra V, Flanders SA, O'Malley M, Malani AN, Prescott HC (2020). Sixty-day outcomes among patients hospitalized with COVID-19. Ann. Intern. Med..

[15] Galvan-Tejada CE (2020). Persistence of COVID-19 symptoms after recovery in mexican population. Int. J. Environ. Res. Public Health.

[16] Garrigues E (2020). Post-discharge persistent symptoms and health-related quality of life after hospitalization for COVID-19. J. Infect..

[17] Horvath L (2020). Smell and taste loss in COVID-19 patients: Assessment outcomes in a Victorian population. Acta Otolaryngol..

[18] Kamal M, Abo Omirah M, Hussein A, Saeed H (2020). Assessment and characterisation of post-COVID-19 manifestations. Int. J. Clin. Pract..

[19] Mandal S (2020). 'Long-COVID': A cross-sectional study of persisting symptoms, biomarker and imaging abnormalities following hospitalisation for COVID-19. Thorax.

[20] Munro KJ, Uus K, Almufarrij I, Chaudhuri N, Yioe V (2020). Persistent self-reported changes in hearing and tinnitus in post-hospitalisation COVID-19 cases. Int. J. Audiol..

[21] Sonnweber T (2020). Persisting alterations of iron homeostasis in COVID-19 are associated with non-resolving lung pathologies and poor patients' performance: A prospective observational cohort study. Respir. Res..

[22] Taquet M, Luciano S, Geddes JR, Harrison PJ (2020). Bidirectional associations between COVID-19 and psychiatric disorder: Retrospective cohort studies of 62 354 COVID-19 cases in the USA. Lancet Psychiatry.

[23] Townsend L (2020). Persistent fatigue following SARS-CoV-2 infection is common and independent of severity of initial infection. PLoS One.

[24] Xiong Q (2021). Clinical sequelae of COVID-19 survivors in Wuhan, China: A single-centre longitudinal study. Clin. Microbiol. Infect..

[25] Alwan NA (2020). Track COVID-19 sickness, not just positive tests and deaths. Nature.

[26] Neufeld KJ (2020). Fatigue symptoms during the first year following ARDS. Chest.

[27] Wostyn P (2021). COVID-19 and chronic fatigue syndrome: Is the worst yet to come?. Med. Hypotheses.

[28] Vink M, Vink-Niese A (2020). Could cognitive behavioural therapy be an effective treatment for long COVID and post COVID-19 fatigue syndrome? lessons from the Qure study for Q-fever fatigue syndrome. Healthcare (Basel).

[29] Lamprecht B (2020). Is there a post-COVID syndrome?. Pneumologe (Berl).

[30] Pallanti S, Grassi E, Makris N, Gasic GP, Hollander E (2020). Neurocovid-19: A clinical neuroscience-based approach to reduce SARS-CoV-2 related mental health sequelae. J. Psychiatr. Res..

[31] Nath A (2020). Long-haul COVID. Neurology.

[32] Beyond Myalgic Encephalomyelitis/Chronic Fatigue Syndrome: Redefining an Illness (2015). Mil. Med..

[33] Proal A, Marshall T (2018). Myalgic encephalomyelitis/chronic fatigue syndrome in the era of the human microbiome: Persistent pathogens drive chronic symptoms by interfering with host metabolism, gene expression, and immunity. Front. Pediatr..

[34] Kingstone T (2020). Finding the ‘right’ GP: A qualitative study of the experiences of people with long-COVID. BJGP Open..

[35] Maury A, Lyoubi A, Peiffer-Smadja N, de Broucker T, Meppiel E (2020). Neurological manifestations associated with SARS-CoV-2 and other coronaviruses: A narrative review for clinicians. Rev. Neurol. (Paris).

[36] Baldini T (2021). Cerebral venous thrombosis and SARS-CoV-2 infection: A systematic review and meta-analysis. Eur. J. Neurol..

[37] Rogers JP (2020). Psychiatric and neuropsychiatric presentations associated with severe coronavirus infections: A systematic review and meta-analysis with comparison to the COVID-19 pandemic. Lancet Psychiatry.

[38] Bacaro V (2020). Insomnia in the Italian population during Covid-19 outbreak: A snapshot on one major risk factor for depression and anxiety. Front. Psychiatry.

[39] Mieczkowska K (2021). Telogen effluvium: A sequela of COVID-19. Int. J. Dermatol..

[40] Zhao YM (2020). Follow-up study of the pulmonary function and related physiological characteristics of COVID-19 survivors three months after recovery. EClinicalMedicine.

[41] Ng CK (2004). Six month radiological and physiological outcomes in severe acute respiratory syndrome (SARS) survivors. Thorax.

[42] Wang Q, Zhang Z, Shi Y, Jiang Y (2013). Emerging H7N9 influenza A (novel reassortant avian-origin) pneumonia: Radiologic findings. Radiology.

[43] Huang Y (2020). Impact of coronavirus disease 2019 on pulmonary function in early convalescence phase. Respir. Res..

[44] Hui DS (2005). Impact of severe acute respiratory syndrome (SARS) on pulmonary function, functional capacity and quality of life in a cohort of survivors. Thorax.

[45] Rouse BT, Sehrawat S (2010). Immunity and immunopathology to viruses: What decides the outcome?. Nat. Rev. Immunol..

[46] Goertz YMJ (2020). Persistent symptoms 3 months after a SARS-CoV-2 infection: The post-COVID-19 syndrome?. ERJ Open Res..

[47] Moore JB, June CH (2020). Cytokine release syndrome in severe COVID-19. Science.

[48] Ngai JC (2010). The long-term impact of severe acute respiratory syndrome on pulmonary function, exercise capacity and health status. Respirology.

[49] Suliman YA (2015). Brief report: Pulmonary function tests: High rate of false-negative results in the early detection and screening of scleroderma-related interstitial lung disease. Arthritis Rheumatol..

[50] Del Rio C, Malani PN (2020). COVID-19-new insights on a rapidly changing epidemic. JAMA.

[51] Jason L, Benton M, Torres-Harding S, Muldowney K (2009). The impact of energy modulation on physical functioning and fatigue severity among patients with ME/CFS. Patient Educ. Couns..

[52] White PD (2011). Comparison of adaptive pacing therapy, cognitive behaviour therapy, graded exercise therapy, and specialist medical care for chronic fatigue syndrome (PACE): A randomised trial. Lancet.

