# Supplementary material for: More than 50 long-term effects of COVID-19: a systematic review and meta-analysis
Source: Sci Rep. 2021 Aug 9;11:16144. doi: 10.1038/s41598-021-95565-8 (PMC8352980; doi:10.1038/s41598-021-95565-8)
Supplement: Supplementary file 2 — Supplementary Information 2. [file 41598_2021_95565_MOESM2_ESM.docx]

**Supplemental Table 1.** *Health states Quality Index variables.*

| **Study** | **1.Population and observation period well defined** | **2.Diagnostic criteria** | **3.Method of case ascertainment** | **4.Administration of measurement protocol** | **Catchment Area** | **Prevalence measure** | **Total (Max: 11)** |
| --- | --- | --- | --- | --- | --- | --- | --- |
| Andrews ^11^ | 1 | 1 | 2 | 3 | 1 | 2 | 10 |
| Carfi ^12^ | 1 | 0 | 1 | 3 | 1 | 2 | 8 |
| Carvalho-Schneider ^13^ | 1 | 1 | 1 | 3 | 2 | 1 | 9 |
| Chopra ^14^ | 1 | 0 | 2 | 1 | 2 | 2 | 8 |
| Galvan-Tejada ^15^ | 1 | 0 | 2 | 3 | 1 | 2 | 9 |
| Garrigues ^16^ | 1 | 1 | 1 | 3 | 1 | 2 | 9 |
| Horvath ^17^ | 0 | 1 | 2 | 3 | 1 | 2 | 9 |
| Kamal ^18^ | 0 | 0 | 3 | 3 | 1 | 2 | 9 |
| Mandal ^19^ | 1 | 1 | 2 | 3 | 2 | 2 | 11 |
| Munro ^20^ | 1 | 0 | 1 | 3 | 1 | 2 | 8 |
| Sonnweber ^21^ | 1 | 1 | 2 | 3 | 2 | 2 | 11 |
| Taquet ^22^ | 1 | 1 | 2 | 2 | 2 | 2 | 10 |
| Tenforde ^3^ | 1 | 0 | 2 | 3 | 2 | 2 | 10 |
| Townsend ^4^ | 1 | 1 | 1 | 3 | 1 | 2 | 9 |
| Xiong ^24^ | 1 | 0 | 2 | 3 | 1 | 2 | 9 |

1. 1) Yes=1, No=0
2. 2) Diagnostic system reported=1, Own system /symptoms described/no system/not specified = 0
3. 3) Community survey/multiple institutions=2, Inpatient/inpatients and outpatients/case registers=1, Not specified=0
4. 4) Administered interview=3, Systematic case note review=2, Chart diagnosis/case records=1,Not specified=0
5. 5) Broadly representative (national or multi-site survey)=2, Small area/not representative (single community, single university)=1, Convenience sampling/ other (primary care sample/treatment group)=0
6. 6) Point prevalence (e.g. one month=2, 12-month prevalence=1, Lifetime prevalence=0
