# Supplementary material for: More than 50 long-term effects of COVID-19: a systematic review and meta-analysis
Source: Sci Rep. 2021 Aug 9;11:16144. doi: 10.1038/s41598-021-95565-8 (PMC8352980; doi:10.1038/s41598-021-95565-8)
Supplement: Supplementary file 3 — Supplementary Information 3. [file 41598_2021_95565_MOESM3_ESM.docx]

**Additional information. Supplementary information.**

**Supplemental Figure 1**. Forest plots of long-term effects of COVID-19.

**Supplementary Table 1.** Health states Quality Index variables.
